# Cdc42 regulates cell polarization and contractile actomyosin rings during terminal differentiation of human erythroblasts

**DOI:** 10.1038/s41598-020-68799-1

**Published:** 2020-07-16

**Authors:** Kumi Ubukawa, Tatsufumi Goto, Ken Asanuma, Yumi Sasaki, Yong-Mei Guo, Isuzu Kobayashi, Kenichi Sawada, Hideki Wakui, Naoto Takahashi

**Affiliations:** 1 Department of Hematology, Nephrology, and Rheumatology, Graduate School of Medicine, Akita University, Akita, Japan; 2 Department of Life Science, Graduate School of Engineering Science, Akita University, Akita, Japan; 3 Division of Radio Isotope, Bioscience Education and Research Support Center, Akita University, Akita, Japan; 4 Hokubukai Utsukushigaoka Hospital, Sapporo, Japan

**Keywords:** Erythropoiesis, Haematopoietic stem cells

## Abstract

The molecular mechanisms involved in the terminal differentiation of erythroblasts have been elucidated by comparing enucleation and cell division. Although various similarities and differences between erythroblast enucleation and cytokinesis have been reported, the mechanisms that control enucleation remain unclear. We previously reported that dynein and microtubule-organizing centers mediated the polarization of nuclei in human erythroblasts. Moreover, the accumulation of F-actin was noted during the enucleation of erythroblasts. Therefore, during enucleation, upstream effectors in the signal transduction pathway regulating dynein or actin, such as cell division control protein 42 homolog (Cdc42), may be crucial. We herein investigated the effects of the Cdc42 inhibitor, CASIN, on cytokinesis and enucleation in colony-forming units-erythroid (CFU-Es) and mature erythroblasts (day 10). CASIN blocked the proliferation of CFU-Es and their enucleation in a dose-dependent manner. Dynein adopted an island-like distribution in the cytoplasm of non-treated CFU-Es, but was concentrated near the nucleus as a dot and co-localized with γ-tubulin in CASIN-treated cells. CASIN blocked the accumulation of F-actin in CFU-Es and day 10 cells. These results demonstrated that Cdc42 plays an important role in cytokinesis, nuclear polarization and nuclear extrusion through a relationship with dynein and actin filament organization during the terminal differentiation of erythroblasts.

## Introduction

Mammalian erythroblasts extrude their nuclei during terminal differentiation. After several cell divisions, erythrocyte progenitors stop proliferation and then condense, polarize, and extrude nuclei^1,2^. Koury et al. previously reported the accumulation of filamentous actin (F-actin) during erythroblast enucleation^3^, and subsequent studies investigated the relationship between erythroblast enucleation and cell division. Although erythroblast enucleation and cytokinesis share a number of similarities, many differences have also been identified.

We previously reported that dynein and microtubule-organizing centers (MTOCs) were key molecules for the formation of the mitotic spindle apparatus and the separation of chromosomes during mitosis in erythroblasts^1^. We also demonstrated that dynein inhibition impaired nuclear polarization, thereby blocking enucleation^1^. After the division site has been selected, the local accumulation of F-actin occurs. We demonstrated that the cleavage furrow in human erythroblasts was composed of non-muscle myosin IIB and actin^2^. Membrane trafficking and membrane fusion to the division site ultimately results in the physical separation of daughter cells^4,5^. A number of crucial signal transduction effectors, including phosphatidylinositol-3-kinase (PI3K)^6^, have been shown to play a role in the enucleation process, mainly through phosphorylation events. Although these findings strongly support a signal transduction system governing cell polarity, dynein accumulation, and the reorganization of actin filaments through mammalian diaphanous-related formin (mDia) 2, the signal transduction pathway involved has not yet been identified^1,7^. These two events, dynein accumulation and the reorganization of actin filaments during the terminal differentiation of human erythroblasts, are regulated by either one or multiple signal transduction pathway(s).

The structure of F-actin at the rear of the translocating nucleus, which is essential for the extrusion of the nucleus, differs between enucleation and cytokinesis. The F-actin structure in erythroblasts is enriched with tropomodulin 1 and non-muscle myosin IIB and extrudes the nucleus^8^. Wolwer et al. previously reported that nuclear extrusion required intracellular calcium signal transduction through the calmodulin and myosin light chain kinase pathway, similar to cytokinesis. However, erythroblasts required extracellular calcium uptake for enucleation, in contrast to cytokinesis^9^. Erythroblast enucleation and asymmetric cell division were also compared. Asymmetric cell division regulators were not required for erythroblast enucleation^10^. In addition, histone deacetylases, proteasomal regulators, mitogen-activated protein kinases, and cyclin-dependent kinases were identified as molecules involved in erythroblast enucleation using a novel chemical screening approach^11^. Although F-actin and non-muscle myosin IIB are involved in the enucleation of erythroblasts, enucleation and cytokinesis are not the same phenomenon.

Cell division control protein 42 homolog (Cdc42) is a member of the Ras homolog (Rho) guanosine triphosphatase (GTPase) family of the Ras superfamily, and, in response to various extracellular stimuli, functions as a binary molecular switch that cycles between guanosine triphosphate (GTP)- and guanosine diphosphate (GDP)-bound active states^12^. Cdc42 regulates the formation of the actin cytoskeleton and microtubule assembly through several effector proteins, such as the IQ motif-containing GTPase-activating protein (IQGAP), p21-activated kinase (PAK), possibly the partitioning-defective (Par)3/Par6 complex, and Wiskott-Aldrich syndrome protein homolog (WASP)/neural-WASP (N-WASP)^13–15^. These regulators function in cell polarization, division, and movement^13^. Toyoshima et al. and Mitsushima et al. also demonstrated that the activation of PI3K by Cdc42 required the accumulation of dynactin, a dynein-binding molecule, for cell polarization in HeLa cells^16,17^. Collectively, these findings indicate that upstream effectors in the signal transduction pathway regulating dynein, such as the small GTPase Cdc42, are crucial during the terminal differentiation of human erythroblasts.

mDia2, a member of the mDia family, is the main functional effector molecule and is regulated by Cdc42 in somatic cells^18^. Watanabe et al. has shown that mDia2 is essential in mammalian cell cytokinesis and that mDia2-induced F-actin forms a scaffold for the contractile ring and then maintains its position in the center of dividing cells^19^. Furthermore, Ji et al. showed that mDia2 plays a significant role in enucleation by affecting the formation of the contractile actomyosin ring in mouse erythroblasts^7^. These findings indicate that Cdc42 regulates the enucleation of human erythroblasts mediated by mDia2. However, Watanabe et al. reported that mDia2-deficient erythroblasts may be enucleated^20^.

Although the basic strategy for a functional analysis of target molecules is the knockdown of the gene expression of target molecules, difficulties are associated with transforming interfering small RNA (siRNA) and/or some expression vectors into primary culture cells, such as erythroblasts. Specific inhibitors are generally used in studies on the functions of target molecules^1,2,21^. A highly specific inhibitor of Cdc42 activity-specific inhibitor, CASIN, 2-[(2,3,4,9-Tetrahydro-6-phenyl-1*H*-carbazol-1-yl) amino] ethanol was recently established^22^.

The results of the present study demonstrated that Cdc42 plays an essential role in cytokinesis and enucleation through the regulation of dynein and actin filament organization during the terminal differentiation of human erythroblasts.

## Results

### Expression of Cdc42 in human CFU-Es and erythroblasts during terminal differentiation

**Figure 1:**
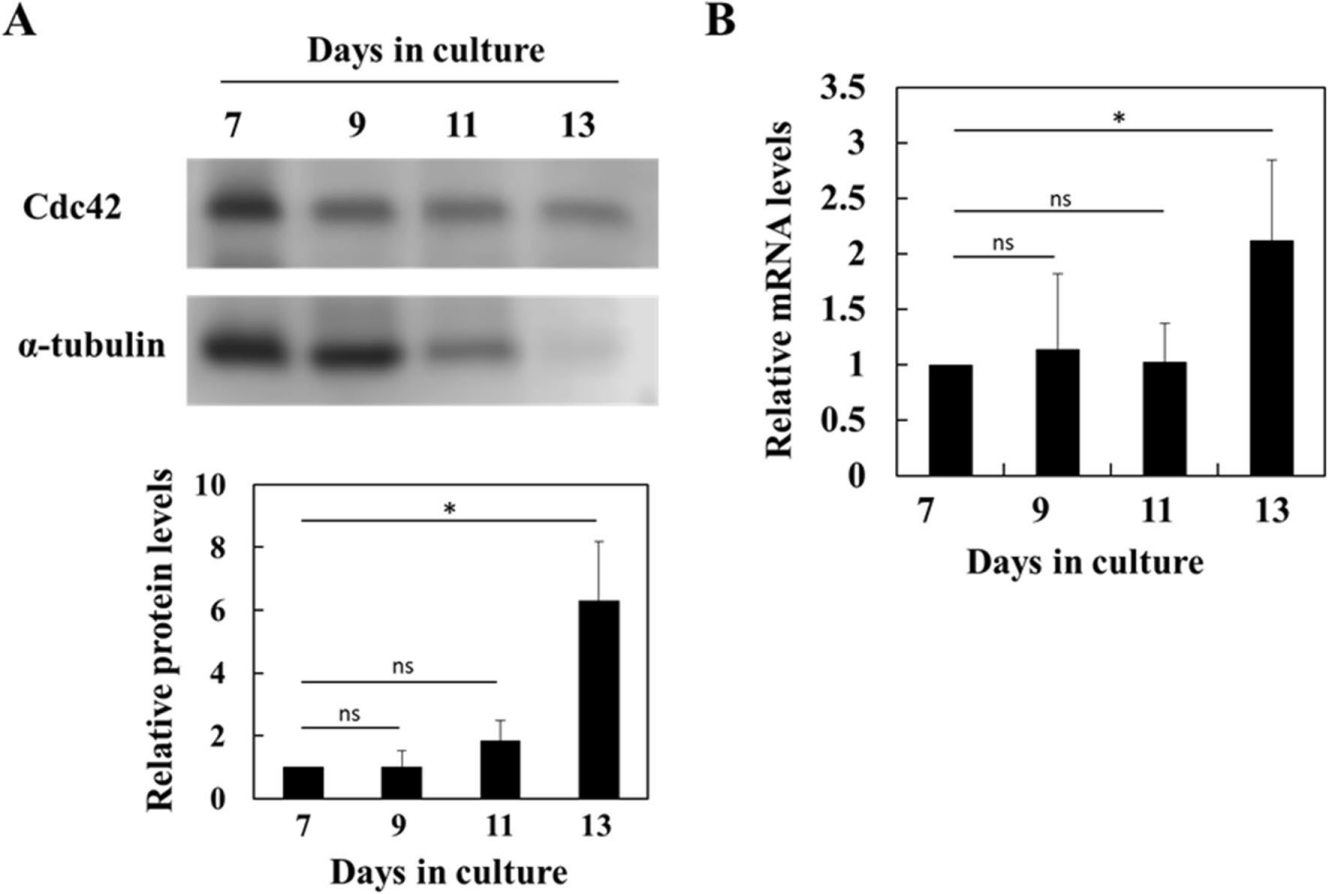
Expression of Cdc42 in CFU-Es and erythroblasts during terminal differentiation. CFU-Es and erythroblasts were harvested at the indicated time points and extracted for an immunoblot or real-time PCR analysis. (**A**) Immunoblot analysis of the expression of Cdc42. α-Tubulin expression was used as a control. A representative result of 3 independent experiments is shown. Day 7 vs. Day 13, *P < 0.05. (**B**) Real-time PCR analysis of *Cdc42*. *28S rRNA* was used as a control. All values were normalized to the value on day 7. Data are shown as the means ± SD of 3 independent experiments. Day 7 vs. Day 13, *P < 0.05.

We examined Cdc42 expression in human CFU-Es and erythroblasts. Regarding protein levels, the immunoblot analysis clearly showed that Cdc42 was constantly expressed in human CFU-Es and erythroblasts, and that the normalized protein levels of Cdc42 increased in erythroblasts during terminal differentiation (Fig. 1A). The expression of α-tubulin, used as a standard for normalization, decreased during terminal differentiation (Fig. 1A). Erythroblasts become smaller as they differentiate and have less protein overall for the same number of cells^23^. We also examined GAPDH as the standard for normalization; however, the results obtained were similar to α-tubulin (data not shown). Original immunoblots were shown in SI Fig. S1. The real-time PCR analysis showed that Cdc42 relative mRNA levels remained unchanged until day 11. On day 13, Cdc42 relative mRNA levels increased (Fig. 1B).

### CASIN inhibited the proliferation and differentiation of human CFU-Es

**Figure 2:**
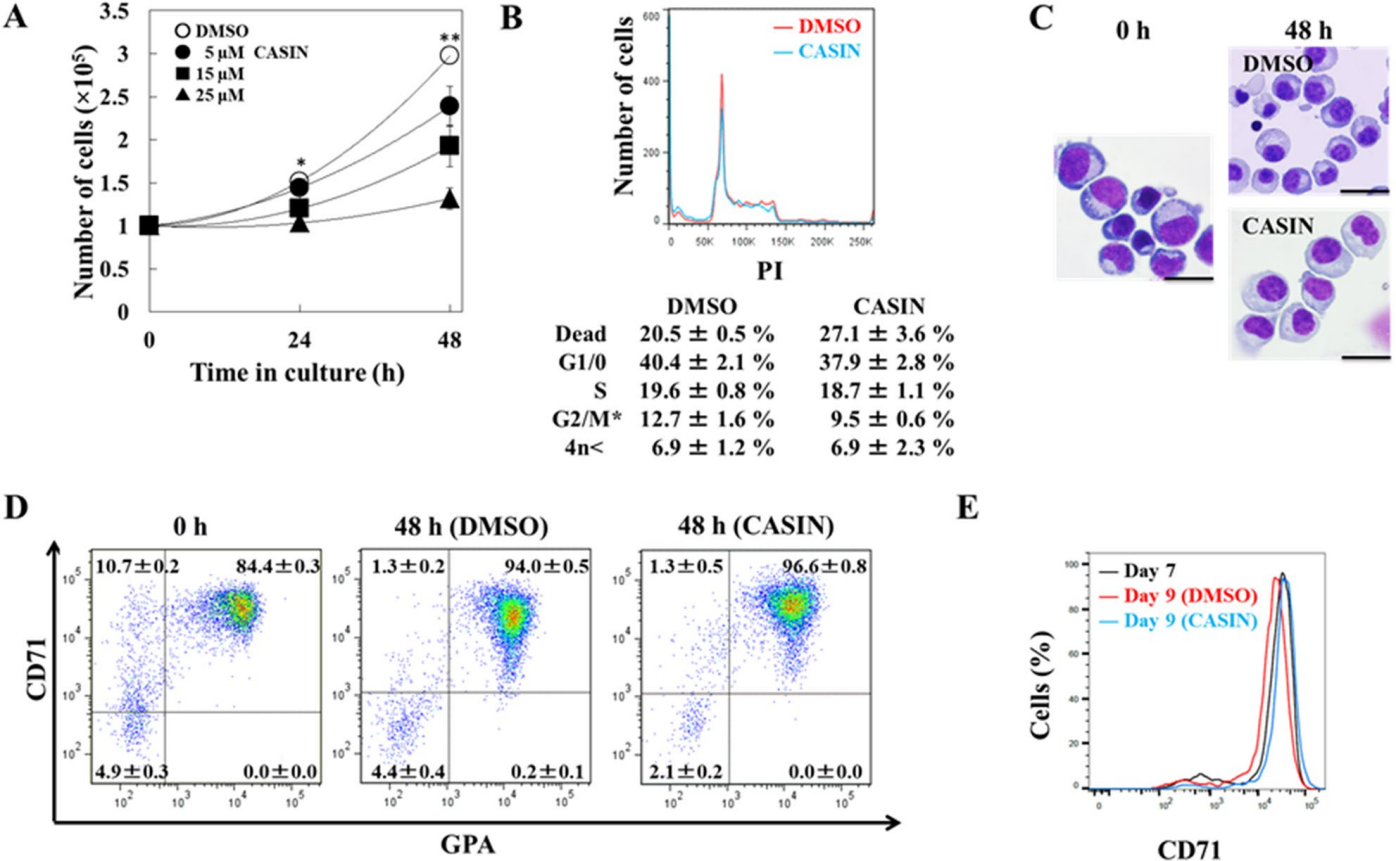
Effects of the inhibition of Cdc42 by CASIN on CFU-E proliferation. (**A**) Day 7 CFU-Es (1 × 10^5^) were cultured in the presence of different concentrations of CASIN (5, 15, or 25 µM) or in its absence (DMSO), and were harvested 24 and 48 h after the addition of CASIN. Data are shown as the means ± SD of 3 independent experiments. DMSO vs. 25 µM CASIN, *P < 0.05 and **P < 0.01. (**B**) Cell cycle analysis of day 8 CFU-Es after being cultured for 24 h in the absence of CASIN (DMSO) (red line) or the presence of 25 µM CASIN (blue line). A representative result from 3 independent experiments is shown as the mean ± SD. DMSO vs. 25 µM CASIN, *P < 0.05. (**C**) Day 7 CFU-Es were cultured for up to 48 h in the absence of CASIN (DMSO) or the presence of 25 µM CASIN. Cells were stained with May–Grünwald–Giemsa reagent. Bar 20 μm. (**D**) Effects of the inhibition of Cdc42 by CASIN on the expression of CD71 and GPA in CFU-Es. Day 7 CFU-Es (1 × 10^5^) were cultured in the presence of 25 µM CASIN or in its absence (DMSO), and were harvested 48 h after the addition of CASIN. Data are shown as the means ± SD of 3 independent experiments. (**E**) Day 7 CFU-Es (black line) were cultured in the presence of 25 µM CASIN (blue line) or in its absence (DMSO, red line), and were harvested 48 h after the addition of CASIN. “Cells (%)” means the relative number of cells with the expression peak of CD71 as 100. Data are shown as the means ± SD of 3 independent experiments.

We investigated the effects of the inhibition of Cdc42 on the proliferation and differentiation of human CFU-Es. The proliferation of CFU-Es was reduced by the CASIN treatment in a concentration-dependent manner (Fig. 2A). The proliferation of erythroblasts was completely inhibited by 25 μM of CASIN, whereas that of DMSO-treated cells as the control was not. The gene and protein expression levels of Cdc42 were not significantly altered in cells cultured with or without CASIN for 48 h (data not shown). We then assessed the effects of CASIN on cell cycle patterns in CFU-Es. After incubating cells for an additional 24 h with CASIN, the cell cycle analysis showed that CASIN reduced the cell fraction in the G2/M phase to significantly less than that with DMSO (9.5 ± 0.6%, 12.7 ± 1.6%, respectively) (Fig. 2B). CASIN-treated cells were larger than control cells at 48 h (Fig. 2C). In the FACS analysis using antibodies to CD71 (transferrin receptor) and GPA, the ratio of cells that expressed these erythroid-specific markers was not altered by the CASIN treatment. CASIN-treated cells were observed to have a cell population accumulated in the upper right as compared with DMSO-treated cells (Fig. 2D). CD71 expression levels were previously shown to be decreased in human erythroblasts during terminal differentiation^24,25^. Interestingly, the expression of CD71 decreased in DMSO-treated cells, but not in CASIN-treated cells, which was similar to the expression of CD71 in cells at 0 h (Fig. 2E). These results showed that the inhibition of Cdc42 by CASIN blocked the proliferation and differentiation of CFU-Es.

### CASIN inhibited the enucleation of human erythroblasts

**Figure 3:**
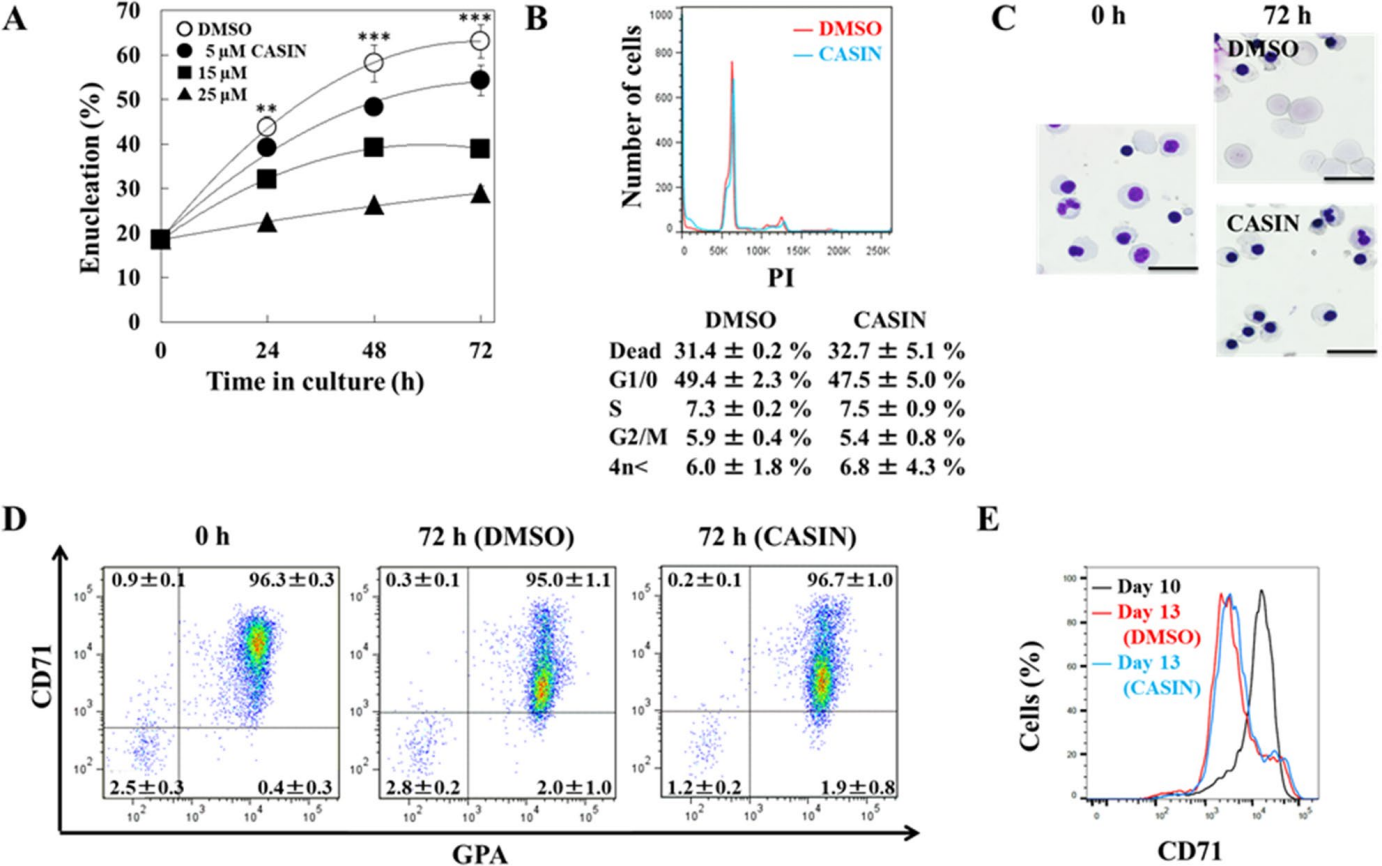
Effects of the inhibition of Cdc42 by CASIN on the ratio of enucleation. (**A**) Day 10 erythroblasts (1 × 10^5^) were cultured in the presence of different concentrations of CASIN (5, 15, or 25 µM) or in its absence (DMSO), and were harvested 24, 48, and 72 h after the addition of CASIN. Data are shown as the means ± SD of 3 independent experiments. DMSO vs. 25 µM CASIN, **P < 0.01 and ***P < 0.001. (**B**) Cell cycle analysis of day 11 erythroblasts after being cultured for 24 h in the absence of CASIN (DMSO) (red line) or the presence of 25 µM CASIN (blue line). A representative result from 3 independent experiments is presented as the mean ± SD. (**C**) Day 10 erythroblasts were cultured for up to 72 h in the absence of CASIN (DMSO) or the presence of 25 µM CASIN. Cells were stained with May–Grünwald–Giemsa reagent. Bar 20 μm. (**D**) Effects of the inhibition of Cdc42 by CASIN on the expression of CD71 and GPA in erythroblasts. Day 10 erythroblasts (1 × 10^5^) were cultured in the presence of different concentrations of 25 µM CASIN or in its absence (DMSO), and were harvested 72 h after the addition of CASIN. Data are shown as the means ± SD of 3 independent experiments. (**E**) Effects of the inhibition of Cdc42 by CASIN on the expression of CD71 in erythroblasts. Day 10 erythroblasts (black line) were cultured in the presence of 25 µM CASIN (blue line) or in its absence (DMSO, red line), and were harvested 72 h after the addition of CASIN. “Cells (%)” means the relative number of cells with the expression peak of CD71 as 100. Data are shown as the means ± SD of 3 independent experiments.

We examined the effects of the inhibition of Cdc42 on the enucleation of erythroblasts. The ratio of enucleation of erythroblasts was reduced by the CASIN treatment in a concentration-dependent manner (Fig. 3A). We then assessed the effects of CASIN on cell cycle patterns in erythroblasts during terminal differentiation. After incubating cells for an additional 24 h with CASIN, the cell cycle analysis showed that none of the phases were altered in the presence of CASIN (Fig. 3B). The enucleation of human erythroblasts was completely inhibited by 25 μM of CASIN, whereas that of DMSO-treated cells was not (Fig. 3C). The gene and protein expression levels of Cdc42 were not significantly changed in cells cultured with or without CASIN for 48 h (data not shown).

In the FACS analysis using antibodies to CD71 and GPA, the ratio of cells that expressed these erythroid specific markers was not changed by the CASIN treatment (Fig. 3D). Additionally, the expression of CD71 was decreased in both DMSO- and CASIN-treated cells (Fig. 3E). These results showed that the inhibition of Cdc42 by CASIN blocked the enucleation, but not differentiation, of erythroblasts from day 10.

**Figure 4:**
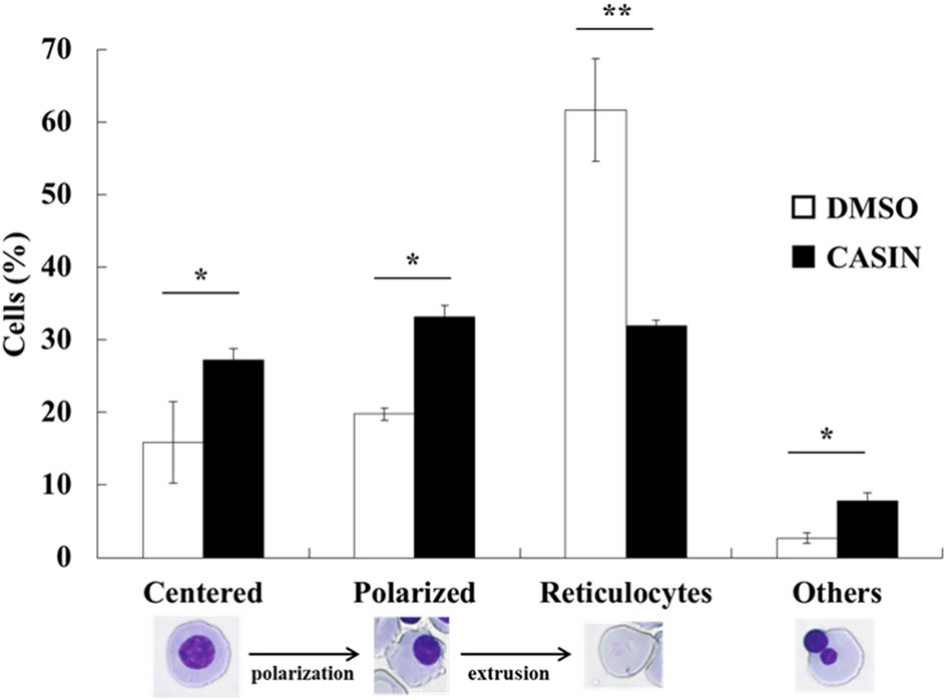
Effects of the inhibition of Cdc42 by CASIN on the ratio of cell polarization. Day 10 erythroblasts (1 × 10^5^) were cultured with or without CASIN, and were harvested 24 h after the addition of CASIN. “Centered”, “Polarized”, “Others”, and “Reticulocytes” were explained in “Methods”. Data are shown as the means ± SD of 3 independent experiments (N = 300). DMSO vs. 25 µM CASIN, *P < 0.05 and **P < 0.01.

We then investigated the effects of CASIN on the positioning of the nucleus in erythroblasts undergoing terminal differentiation. After incubating day 10 cells for an additional 24 h with CASIN, the ratio of centered, polarized, and others significantly increased over that in DMSO-treated cells. In contrast, the ratio of reticulocytes in CASIN-treated cells was lower than that in DMSO-treated cells (Fig. 4). This result showed that the inhibition of Cdc42 by CASIN blocked the polarization and extrusion of nuclei in human erythroblasts during terminal differentiation.

### Effects of inhibiting Cdc42 by CASIN on the localization of Cdc42, mDia2, and actin filaments in human CFU-Es and erythroblasts during terminal differentiation

**Figure 5:**
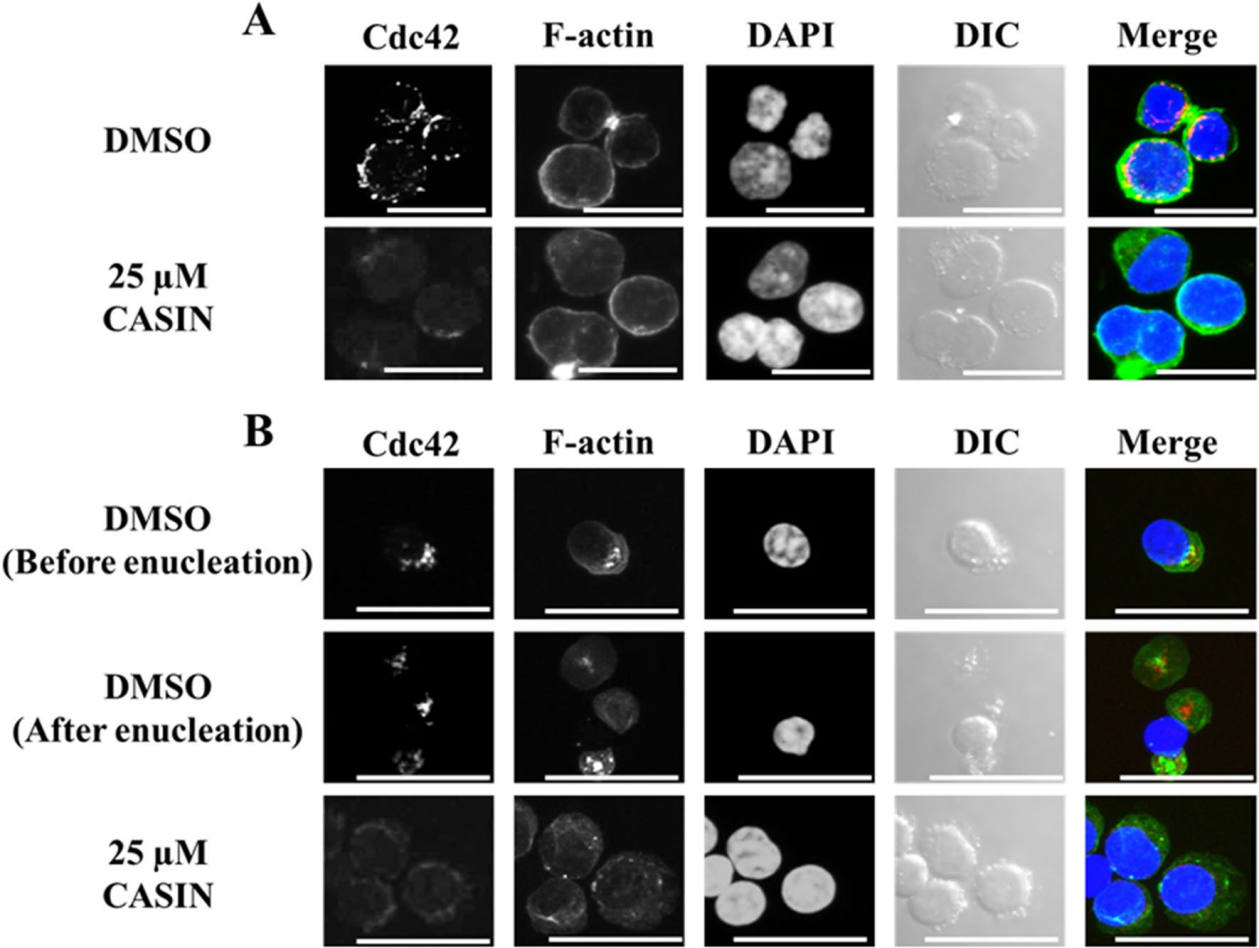
Distribution of Cdc42 and F-actin in CFU-Es and erythroblasts with or without the CASIN treatment. Confocal microscopy of (**A**) CFU-Es (day 8 cells) and (**B**) erythroblasts (day11 cells) stained by Cdc42 (red), phalloidin (green), and DAPI (blue), and differential interference contrast microscopic images (DIC) are shown. These are entire confocal stacks. DMSO and 25 µM CASIN denote day 7 or day 10 cells, respectively, treated for 24 h without and with 25 µM CASIN. Bar 20 µm.

In CFU-Es (day 8 cells), Cdc42 was localized beside the contractile actomyosin ring (Fig. 5A). However, in cells incubated with CASIN for an additional 24 h, Cdc42 was not localized in the specific contractile actomyosin ring point and, thus, this ring was not formed (Fig. 5A). In day 11 cells, Cdc42 was localized beside the accumulation of F-actin, similar to CFU-Es (Fig. 5B). Furthermore, after enucleation, Cdc42 was localized inside reticulocytes. However, in cells incubated for an additional 24 h with CASIN, Cdc42 was not localized near the nucleus, and F-actin did not accumulate (Fig. 5B).

**Figure 6:**
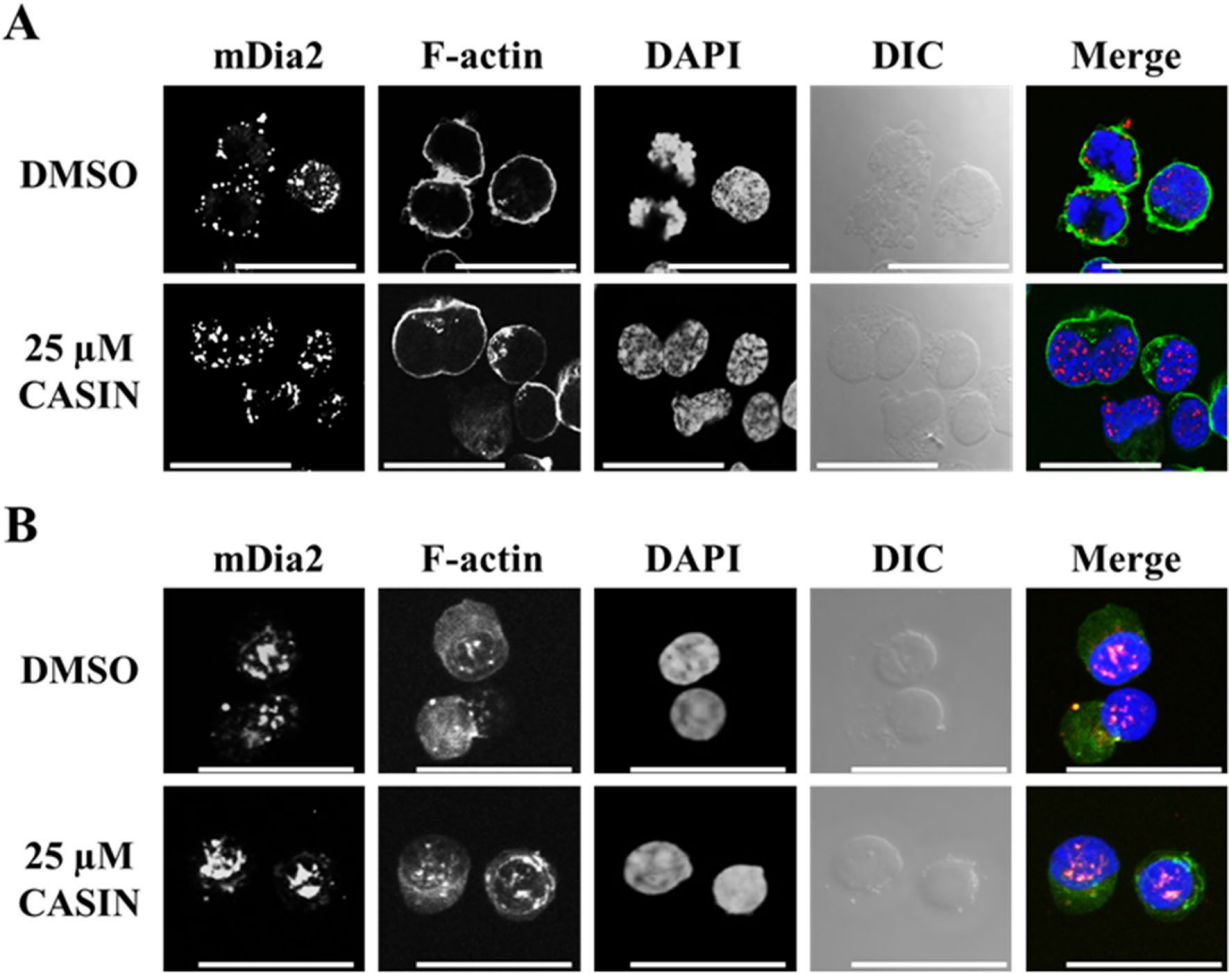
Distribution of mDia2 and F-actin in CFU-Es and erythroblasts with or without the CASIN treatment. Confocal microscopy of (**A**) CFU-Es (day 8 cells) and (**B**) erythroblasts (day 11 cells) stained by mDia2 (red), phalloidin (green), and DAPI (blue) and DIC are shown. These are entire confocal stacks. DMSO and 25 µM CASIN denote day 7 or day 10 cells, respectively, treated for 24 h without and with 25 µM CASIN. Bar 20 µm.

In mouse erythroblasts, mDia2 plays a significant role in enucleation by influencing the formation of the contractile actomyosin ring^7^. We also examined the localization of mDia2 in CFU-Es or erythroblasts. In day 8 cells, mDia2 was localized in the cytoplasm near the cell membrane in dividing cells and in the nucleus of interphase cells (Fig. 6A). Among cells incubated for an additional 24 h with CASIN, the number of mitotic phase cells was small, and mDia2 was also localized in the nucleus (Fig. 6A). In day 11 cells, mDia2 was localized in the nucleus regardless of the treatment with CASIN (Fig. 6B). These results suggest that Cdc42 is involved in the formation of the contractile actomyosin ring in erythroblasts, and that mDia2 as a downstream effector of Cdc42 is associated with the proliferation of erythroblasts, while mDia2 does not appear to be associated with enucleation.

### Effects of the inhibition of Cdc42 by CASIN on the localization of dynein and γ-tubulin in human CFU-Es and erythroblasts during terminal differentiation

**Figure 7:**
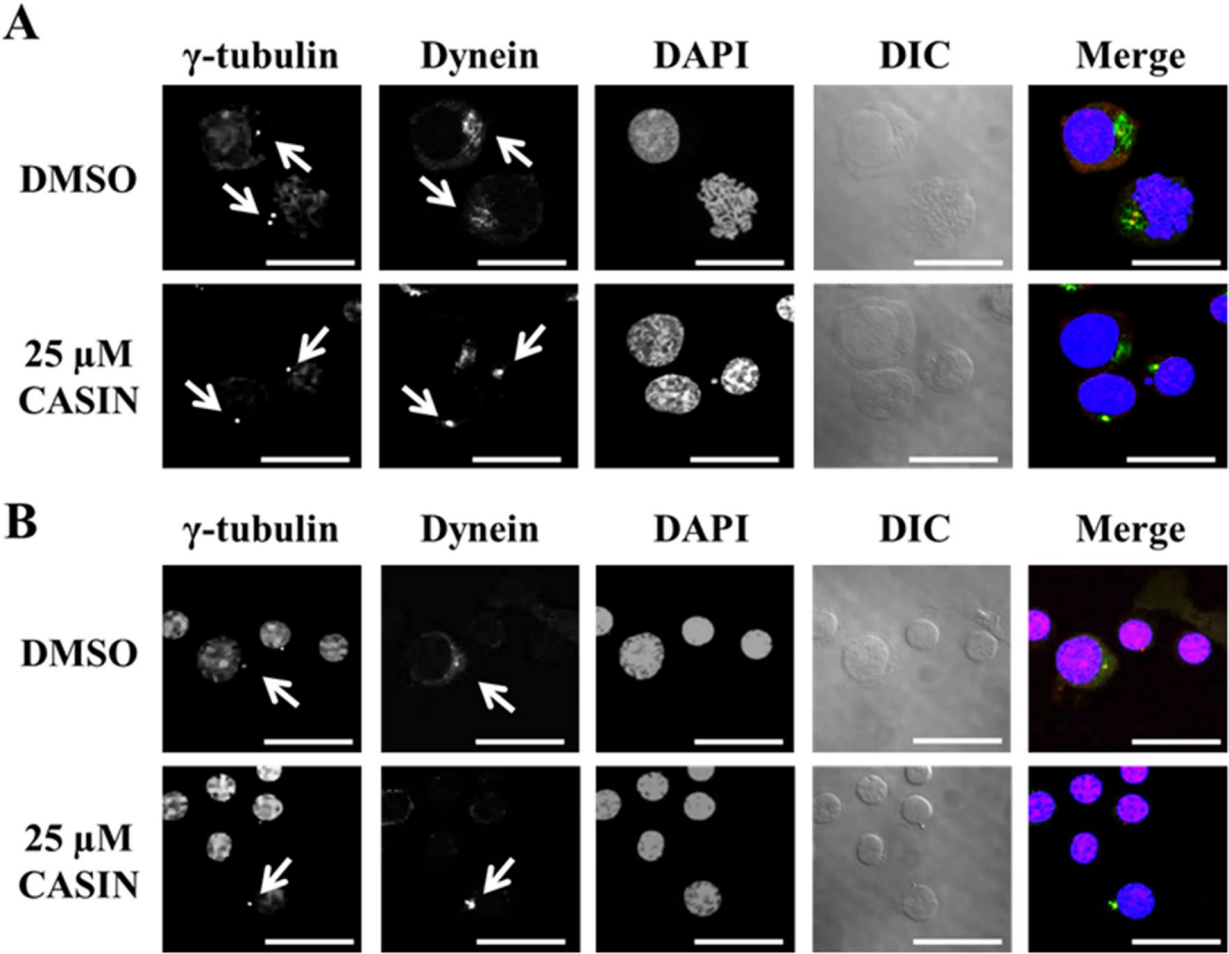
Distribution of γ-tubulin and dynein in CFU-Es and erythroblasts with or without the CASIN treatment. Confocal microscopy of (**A**) CFU-Es (day 8 cells) and (**B**) erythroblasts (day 11 cells) stained by γ-tubulin (red), dynein (green), and DAPI (blue) and DIC are shown. These are entire confocal stacks. DMSO and 25 µM CASIN denote day 7 or day 10 cells, respectively, treated for 24 h without and with 25 µM CASIN. Bar 20 µm.

In CFU-Es, γ-tubulin was located in the center of cells, while dynein had an island-like distribution in the cytoplasm of non-treated CFU-Es. However, in CASIN-treated cells, dynein was localized near the nucleus in a dot pattern and was co-localized with γ-tubulin (Fig. 7A). In day 11 cells, dynein had an island-like distributed in the cytoplasm, similar to day 8 cells. On the other hand, in CASIN-treated cells, dynein was localized near the nucleus in a dot pattern and was co-localized with γ-tubulin (Fig. 7B). These results indicate that Cdc42 regulates the motion of dynein in erythroblasts.

## Discussion

In the present study, we investigated the expression and localization of Cdc42 in human erythroblasts during terminal differentiation. Furthermore, the inhibition of Cdc42 activity by CASIN blocked the proliferation and differentiation of CFU-Es and enucleation of erythroblasts. A key result was that the inhibition of Cdc42 blocked both the cell polarization and formation of contractile rings in human erythroblasts during terminal differentiation. These results suggest that Cdc42 signaling transduction is constantly used for the maturation process of human erythroblasts.

A previous study by Yang et al. demonstrated that a Cdc42 deficiency caused anemia and splenomegaly accompanied by decreased burst-forming units-erythroid (BFU-Es) and CFU-Es in mice^26^. Their findings indicated that a Cdc42 deficiency causes a block in the early stage of erythropoiesis in mice. The present results suggested that the inhibition of Cdc42 blocked both the proliferation and differentiation of CFU-Es. In contrast, to the best of our knowledge, the functional roles of Cdc42 in the late stage of erythropoiesis currently remain unknown. Our results showed that the inhibition of Cdc42 impaired both the polarization and extrusion of the nucleus in the late stage of human erythroblasts, suggesting that Cdc42 acts on both the dynein–tubulin system and actin–myosin system in human erythroblasts.

In the present study, we examined the effects of the inhibition of Cdc42 on the localization of dynein in human erythroblasts. Tuncay et al. reported that junctional adhesion molecule-A (JAM-A) regulated dynein localization mediated by Cdc42 to control planar spindle orientation during mitosis in epithelial cells^27^. Their findings showed that JAM-A triggered the transient activation of Cdc42 and PI3K, generated a gradient of PtdIns(3,4,5)P3 at the cortex, and regulated the formation of the cortical actin cytoskeleton during mitosis. When the JAM-A was inhibited by siRNA, dynactin that forms a complex with dynein localization at the cortex is reduced, the mitotic spindle apparatus is misaligned, and epithelial morphogenesis in a three-dimensional culture is compromised^27^. The present results showed that the localization of dynein was changed from an island-like distribution in the cytoplasm to near the nucleus in a dot pattern and was co-localized with γ-tubulin by the inhibition of Cdc42 in human CFU-Es and erythroblasts. These results are consistent with the localization of dynactin at the cortex being reduced and only being near MTOCs in JAM-A knockdown cells^27^. Collectively, these findings and the present results imply that signaling through Cdc42 stops the motion of the dynein–dynactin complex, and, thus, further studies are required.

To the best of our knowledge, the localization of Cdc42 during human erythropoiesis has not yet been examined. This is the first study to characterize the localization of Cdc42 in human erythroblasts. We confirmed that Cdc42 was localized at the position at which the accumulation of F-actin occurred in CFU-Es and erythroblasts. In somatic cells, the localization and activity of Cdc42 during cytokinesis has not yet been examined in detail in animal cells^28^. Previous studies reported that RhoA, which is similar to Cdc42, was important for forming contractile actomyosin rings; however, these findings are controversial^28^. The present results imply that Cdc42 plays an important role in the formation of contractile actomyosin rings in CFU-Es and erythroblasts.

We showed that during the cell division of CFU-Es, the localization of mDia2 changed from the nucleus to the cytoplasmic membrane. This result is in agreement with previous findings obtained using human and mouse cells^29,30^. Moreover, we found that the localization of mDia2 remained unchanged in the nucleus during the enucleation of erythroblasts. Ji et al. demonstrated that the percentage of enucleated erythroblasts in mDia2 shRNA-treated cells in mice decreased, and suggested the involvement of mDia2 in enucleation^7^. On the other hand, Watanabe et al. demonstrated that mDia2-deficient mouse erythroid cells differentiated normally, albeit in a delayed manner, but exhibited failed cytokinesis with the decreased accumulation of F-actin in the cleavage furrow during late differentiation from proerythroblasts^20^. mDia2-deficient mouse erythroblasts enucleate their nuclei. These findings suggest blocked enucleation by the inhibition of mDia2 in mouse erythroid cells due to failed cytokinesis during erythroid differentiation. The present results appear to support Watanabe’s findings. Additionally, the inhibition of Cdc42 changed the localization of mDia2 in CFU-Es during cell division, but not the enucleation of erythroblasts. These results suggest that mDia2 as a downstream molecule of Cdc42 was involved in at least the proliferation of CFU-Es, but not in the enucleation of erythroblasts. To confirm this, further information on whether mDia2 is essential for the enucleation of human erythroblasts is needed. It is important for us to establish a gene transfer technique in human erythroblasts.

Pathologically, an abnormality in Cdc42 causes a clinically heterogeneous group of phenotypes characterized by variable growth dysregulation, facial dysmorphism, and neurodevelopmental, immunological, and hematological anomalies^31^. A previous study reported that hematological anomalies include lymphatic and platelet anomalies^31^. However, to the best of our knowledge, erythrocytic anomalies, such as anemia, attributed to Cdc42 abnormalities have not yet been reported. In mice, a Cdc42 deficiency is a fatal symptom with anemia^26^, and this may also be the case in humans.

In conclusion, we herein demonstrated that Cdc42 is important in human erythropoiesis. Furthermore, the present results indicate that Cdc42 plays an important role in both nuclear polarization and nuclear extrusion through the control of dynein and actin filament organization during the terminal differentiation of human erythroblasts.

## Methods

### Reagents and inhibitors

Bovine serum albumin (BSA), Iscove’s Modified Dulbecco’s Medium (IMDM), and propidium iodide (PI) were purchased from Sigma-Aldrich (St. Louis, MO, USA). Fetal calf serum (FCS) was from HyClone Laboratories Inc. (Logan, UT). Penicillin and streptomycin were from Flow Laboratories. Insulin was from Wako Pure Chemical Industries (Osaka, Japan). Interleukin-3 (IL-3) and stem cell factor (SCF) were from Kirin Brewery Co., Ltd. (Tokyo, Japan). Erythropoietin (EPO) and granulocyte colony-stimulating factor (G-CSF) were from Chugai Pharmaceutical Co. (Tokyo, Japan). Vitamin B_12_ was from Eisai Co., Ltd. (Tokyo, Japan), and folic acid was from Takeda Pharmaceutical Co., Ltd. (Osaka, Japan). Triton X-100 was from Wako Pure Chemical Industries (Osaka, Japan). MACS MicroBeads for indirect magnetic labeling were from Miltenyi Biotec (Bergisch-Gladbach, Germany). Normal mouse and goat sera as well as rabbit immunoglobulins were from Cell Signaling Technology (Beverly, MA, USA). CASIN, 2-[(2,3,4,9-Tetrahydro-6-phenyl-1*H*-carbazol-1-yl) and the antibodies used in the present study are listed in Table 1.

**Table 1:** Antibodies used in immunoblot analyses or immunocytochemical staining.

Antigen^a^	Host animal/polyclonal or monoclonal^b^	Vendor information
α-Tubulin	Mouse/mono	Sigma-Aldrich-T9026
γ-Tubulin	Mouse/mono	Abcam-ab11316
Cdc42	Rabbit/poly	Millipore-07–1,466
Cdc42	Mouse/mono	Santa Cruz-sc-8401
Dynein	Rabbit/mono	Abcam-ab157468
mDia2	Mouse/mono	Santa Cruz-sc-293288
Alexa Fluor 488 mouse IgG	Goat/poly	Thermo Fisher-A-11001
Alexa Fluor 488 rabbit IgG	Goat/poly	Thermo Fisher-A-11008
Alexa Fluor 546 mouse IgG	Goat/poly	Thermo Fisher-A-11003
Alexa Fluor 546 rabbit IgG	Goat/poly	Thermo Fisher-A-11010
HRP-linked mouse IgG	Goat/poly	KPL-074-1809
HRP-linked rabbit IgG	Goat/poly	Cell Signaling-7074

^a^The amino acid sequences of all antigens were derived from human resources.

^b^Poly or mono indicates a polyclonal or monoclonal antibody, respectively.

### Cell preparations

G-CSF-mobilized human peripheral blood CD34^+^ cells were purified from healthy volunteers as described previously and stored in liquid nitrogen until required^32^. Informed consent was obtained from all subjects prior to their entry into the present study, which was pre-approved by the Akita University Graduate School of Medicine Committee for the Protection of Human Subjects, according to the Declaration of Helsinki^21^. To generate erythroid progenitor cells, CD34^+^ cells were thawed out and prepared for culture as previously described^1,2,33^. Cells were cultured in IMDM erythroid medium containing 20% FCS, 10% heat-inactivated pooled human AB serum, 1% BSA, 10 μg/mL insulin, 0.5 μg/mL vitamin B_12_, 15 μg/mL folic acid, 50 nM β-mercaptoethanol, 50 U/mL penicillin, and 50 μg/mL streptomycin and supplemented with 50 ng/mL IL-3, 50 ng/mL SCF, and 2 IU/mL EPO. Cells were maintained in an incubator at 37 °C and 5% CO_2_. On day 7 of the culture (day 7), cells were harvested and washed three times with IMDM containing 0.3% BSA and stored at 4 °C until further use. The maturation stage of day 7 cells was similar to that of CFU-Es, as reported previously^1,2,33^. Day 7 cells were hereafter referred to as CFU-Es. Aliquots of CFU-Es were cultured in erythroid medium with EPO alone without β-ME, SCF, or IL-3 to induce differentiation and supplemented with or without CASIN^22^. DMSO, a solvent of CASIN, was used as a control. The final concentration of DMSO was 0.025%, which was markedly lower than the concentration that is toxic to human erythroblasts^2^. Cells were harvested at various time points, washed three times with IMDM containing 0.3% BSA, resuspended in IMDM containing 0.3% BSA, and stored at 4 °C until further use.

To evaluate enucleation, cells were spun down onto slides using Cytospin 3 (Shandon Lipshaw Inc., Pittsburgh, PA, USA) and stained with May–Grünwald–Giemsa. Enucleation was defined as the expulsion of the nucleus to the outside of the reticulocyte. Reticulocytes still in contact with expelled nuclei or with a thin connecting strand between the reticulocyte and nucleus were regarded as the latest enucleated cells. The enucleation fraction was calculated using the formula [erythrocytes/(erythrocytes + erythroblasts)] × 100 (%) after counting 300 cells, including erythrocytes and erythroblasts, in each slide. Triplicate cultures were used at each time point. Yield and viability were assessed based on dye exclusion using 0.2% trypan blue^1,2,33^.

### Real-time PCR analysis

Real-time PCR was performed as previously described^1,21^. Briefly, total RNA was extracted from 2 × 10^5^ cultured cells per sample using the RNeasy Mini kit (QIAGEN, Hilden, Germany). Extracted RNA was reverse-transcribed using the PrimeScript 1st strand cDNA Synthesis Kit (TAKARA Bio Inc., Shiga, Japan). The resultant cDNA was then subjected to real-time PCR using LightCycler 480 SYBR Green I Master (Roche Applied Science, Mannheim, Germany). PCR primer sequences: *CDC42* forward primer 5′-CTGTCAAGTATGTGGAGTGTTCTGC-3′ and reverse primer 5′-CTCTTCTTCGGTTCTGGAGGCT-3′; *28S rRNA* forward primer 5′-TGGGTTTTAAGCAGGAGGTG-3′ and reverse primer 5′-CCAGCTCACGTTCCCTATTA-3′. Relative gene expression levels were normalized with the *28S rRNA* gene as previously described^34^. Each sample was amplified in triplicate.

### Immunoblot analysis

An immunoblot analysis was performed as previously described^1,2,21^. The primary and secondary antibodies used in the present study are listed in Table 1. ECL Select Western Blotting Detection Reagent (GE Healthcare, Buckinghamshire, UK) was used for color development. The intensities of immunoreactive protein bands were quantified using c-DiGit (LI-COR, Nebraska, USA). The relative expression levels of the proteins analyzed were normalized with α-tubulin expression^1,2,21^.

### Flow cytometry

Flow cytometry was performed as previously described^1^. Briefly, cells collected from cultures were washed twice with IMDM containing 0.3% BSA, and incubated with a phycoerythrin-conjugated mouse monoclonal antibody to human CD71 (transferrin receptor) (BD Biosciences, Franklin Lakes, NJ, USA) and fluorescein isothiocyanate-conjugated mouse monoclonal antibody to human GPA (Dako, Santa Clara, CA, USA). Cells were then washed twice with 10 mM sodium phosphate buffer, pH 7.4 and 0.15 M NaCl (phosphate-buffered saline: PBS) containing 0.5% BSA, and then analyzed using FACSCanto II (BD Biosciences) and FlowJo (BD Biosciences). Cell cycle distribution was performed as previously described^1,2^.

### Morphological and immunochemical analyses of cells cultured with and without inhibitors

Cells were classified based on whether the nucleus was localized at the center of cells (centered); spherical cells contained a condensed nucleus located to one side, near the plasma membrane (polarized), or were enucleated (reticulocytes). Other cell types, such as multinucleate cells and cells with condensed apoptotic nuclei, were classified as “others”. Images of cells were classified as previously described^1^. The fractions of each cell type were calculated as described above. Results are presented as the mean of 3 independent experiments, mean ± SD.

### Confocal microscopy

The immunochemical distributions of Cdc42, F-actin, dynein, γ-tubulin, α-tubulin, and mDia2 were assessed using confocal microscopy, as described above. Day 7 or day 10 cells were cultured in the presence or absence of 25 μM CASIN.

Conventional immunocytochemical staining was performed as described previously^1,2^. Fluorescent staining was imaged using two confocal laser scanning microscopes (LSM780, Carl Zeiss Microscope Systems, Germany) equipped with 100× objective lenses and 10× camera lenses (Carl Zeiss Microscope Systems) at zoom 3, as reported elsewhere^1^. Fluorochromes were excited using an argon laser at 488 nm for Alexa 488. Detector slits were configured to minimize cross talk between channels and processed using ZEN2012 Ver 3.2 (ZEISS).

### Statistical analysis

Statistical analyses were performed using the Student’s *t* test for parametric data and the Mann–Whitney *U* test for non-parametric data. *P* values < 0.05 were considered to be significant in all analyses.

## Supplementary information

Supplementary file1 (DOCX 265 kb)
