# Supplementary material for: Cdc42 regulates cell polarization and contractile actomyosin rings during terminal differentiation of human erythroblasts
Source: Sci Rep. 2020 Jul 16;10:11806. doi: 10.1038/s41598-020-68799-1 (PMC7366696; doi:10.1038/s41598-020-68799-1)
Supplement: Supplementary file 1 — Supplementary file1 (DOCX 265 kb) [file 41598_2020_68799_MOESM1_ESM.docx]

**Cdc42 regulates cell polarization and contractile actomyosin rings**

**during terminal differentiation of human erythroblasts**

Kumi Ubukawa^1#^, Tatsufumi Goto^2#^, Ken Asanuma^3^, Yumi Sasaki^2^, Yong-Mei Guo^1^,

Isuzu Kobayashi^1^, Kenichi Sawada^4^, Hideki Wakui^2^, and Naoto Takahashi^1^

^1^Department of Hematology, Nephrology, and Rheumatology, Master’s Course at the Graduate School of Medicine, Akita University, Akita, Japan

^2^Department of Life Science, Graduate School of Engineering Science, Akita University, Akita, Japan

^3^Division of Radio Isotope, Bioscience Education and Research Support Center, Akita University, Akita, Japan

^4^Hokubukai Utsukushigaoka Hospital, Sapporo, Japan.

**Supplementary Figure**

**
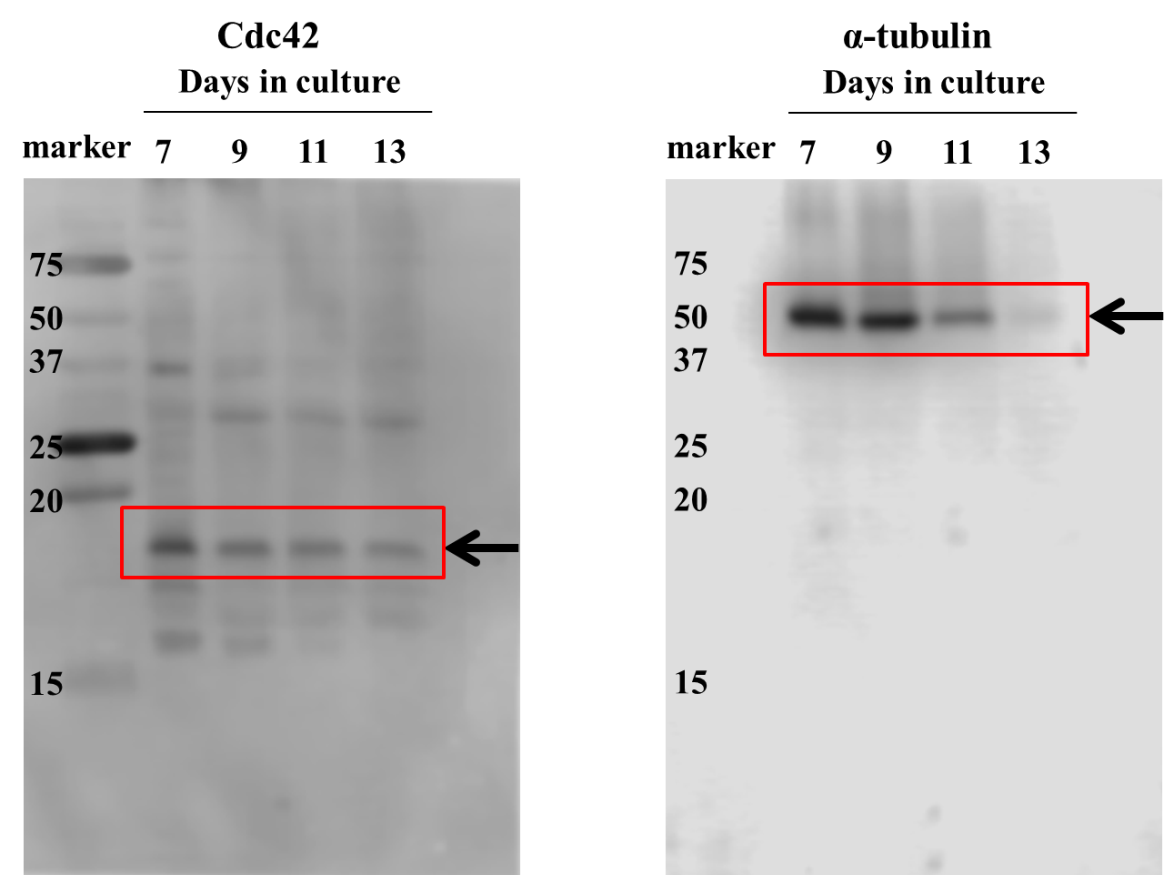
**

**Supplementaly Fig. S1 Original immunoblots related to Fig. 1A**
